# A massive hemorrhagic pleural effusion does not exclude the diagnosis of tuberculosis: a case report

**DOI:** 10.4076/1757-1626-2-8707

**Published:** 2009-07-31

**Authors:** Charalampos Mermigkis, Antony Kopanakis, Kostas Psathakis, Napoleon Karagiannidis, Manolis Kastanakis, Michael Patentalakis, Anastasia Amfilochiou, Georgios Patentalakis, Vlassis Polychronopoulos

**Affiliations:** 1 Department of Pneumonology, Army General HospitalAthensGreece; 2 Third Department of Pneumonology, Sismanoglio General District HospitalAthensGreece

## Abstract

We report a case of an immunocompetent 18-year-old man with a massive hemorrhagic, exudative, lymphocytic pleural effusion. Blind transthoracic pleural biopsy showed granuloma formation, while the pleural fluid culture was positive for *Mycobacterium tuberculosis*, confirming the diagnosis of primary tuberculous pleuritis. A massive hemorrhagic pleural effusion is extremely rare in tuberculosis, but tuberculosis is a very protean disease and should always be included in the differential diagnosis of pleural effusions

## Case presentation

An 18-year-old Greek male, previously healthy, non- smoker, was referred to our hospital because of chest pain and fever, which had been started about two weeks previously. He had been treated with broad spectrum antibiotics as an outpatient without response. His past medical history was unremarkable.

On admission, he had a temperature of 38^°^C, pulse rate 100 beats/min. Physical examination revealed dullness on percussion and absent of breath sounds at the left hemithorax. Laboratory blood tests revealed a reduced hematocrit (29.5%) and elevated erythrocyte sedimentation rate (125 mm/h) and C-reactive protein (8.6 mg/dl). Arterial blood gases showed hypoxemia (PO_2_ = 69 mmHg on room air). The rest of the clinical and laboratory examinations were normal. Chest radiography and computed tomography (CT) scan showed a large (more than 2/3 of the left hemithorax was opacified in admission chest radiography), homogenous, non-loculated pleural effusion on the left, with no parenchymal lesions. Flexible bronchoscopy was negative for endobronchial mass, and showed mild edema of the left lower lobe bronchial mucosa, probably due to extrinsic compression. Smears and cultures for *Mycobacterium tuberculosis* taken from bronchial secretions, and bronchoalveolar lavage (BAL) were negative. Cytology and Gram stain of the above specimens were also negative. Diagnostic thoracocentesis revealed an exudative fluid with hemorrhagic appearance (hematocrit 8%, complete cell count 680/μL with 18% neutrophils and 75% lymphocytes). Pleural fluid Gram stain, acid-fast bacilli (AFB) smears and cytology were all negative. Pleural fluid adenosine deaminase (ADA) level was 70 IU/L. PPD skin test (0.1 ml of PPD-RT 23, 2 TU) was positive (15 mm induration). Blind needle biopsy of the pleura showed the presence of noncaseating granulomas. Based on these features the diagnosis of tuberculous (TB) pleuritis was established and the patient was started on a 6 month anti-TB regimen with 4 drugs (Isoniazid, Rifampicin, Ethambutol and Pyrazinamide). Clinical improvement was noted gradually. Pleural fluid cultures became positive for M. tuberculosis several weeks later.

## Discussion

To the best of our knowledge this is the first reported case of a massive hemorrhagic pleural effusion due to TB.

TB, the single most frequent infectious cause of death worldwide, is also a major cause of pleural effusion. Primary TB pleuritis most usually manifests as an acute or subacute illness with chest pain, cough, fever or dyspnea being the common symptoms. The effusion is usually an exudate with a lymphocyte predominance and is almost always unilateral and small to moderate in size [1-4].

An acute illness with pleuritic pain, fever and exudative lymphocytic pleural effusion in a young previously healthy man with a positive PPD skin test, in a country with relatively high incidence of infection is strongly suggestive of TB [1,3]. However, if the aspirated pleural fluid during thoracentesis is bloody, especially in a large size effusion the diagnosis of TB is considered unlikely [3-5]. In the literature, only four cases of hemorrhagic TB pleuritis have been reported and none of them was massive [6-8]. This has led to the “conventional” knowledge that a hemorrhagic pleural effusion most commonly suggests one of the three following diagnoses: malignant disease, trauma or pulmonary embolization. Less frequent diagnoses for a hemorrhagic pleural effusion may include pneumonia, hematologic disorders or endomitriosis [3].

Currently, the diagnosis of TB pleuritis is practically established when the ratio of lymphocytes to neutrophils in the pleural fluid is > 0.75 and the ADA is > 70 IU/L [2,9]. The diagnostic yield of pleural fluid culture for M. Tuberculosis is generally low and is delayed. Pleural biopsy has the best diagnostic yield for TB pleuritis, but it is rarely necessary when the patient meets the above mentioned criteria [10]. Our patient had a clinical course and pleural fluid characteristics highly suspicious for TB. However, in the presence of a bloody fluid, we preferred to confirm the diagnosis with a pleural biopsy first, before initiating an anti-TB therapy (therapeutic criterion in this case, since the cultures were unknown yet). This case remind us that TB has been recognised as a very protean disease (“one thousand faces disease”), and should always be included in the differential diagnosis of a patient with a lymphocytic pleural exudate, being it hemorrhagic or not, small or massive.

## Abbreviations

ADA: adenosine deaminase
AFB: acid-fast bacilli
BAL: bronchoalveolar lavage
CT: computed tomography
TB: tuberculous

## References

[bib-001] Valdés L, Alvarez D, San José E, Penela P, Valle JM, García-Pazos JM, Suárez J, Pose A (1998). Tuberculous pleurisy: a study of 254 patients. Arch Intern Med.

[bib-002] Light RW (2001). Pleural diseases.

[bib-003] Fraser R, Pare P, Fraser R, Pare P (1994). Specific Causes of Pleural Effusion: Mycobacterium Tuberculosis. Diseases of the Chest.

[bib-004] Siebert AF, Haynes J, Middleton R (1991). Tuberculous pleural effussion: 20-year experience. Chest.

[bib-005] Gopi A, Madhavan S, Sharma S, Sahn ST (2007). Diagnosis and Treatment of Tuberculous Pleural Effusion in 2006. Chest.

[bib-006] Murin S, Moritz E (1996). Bilateral Tuberculous Pleural Effusions with markedly Different Characteristics. Chest.

[bib-007] Renert WA (1971). Hemorrhagic pleural effusion: an unusual finding in tuberculous pleurisy. Conn Med.

[bib-008] Papamichalopoulos A, Mavrou I, Kostadima E, Trakopoulos G (1999). Bilateral haemorrhagic tuberculous pleuritis in an 81-year-old female. Int J Tuberc Lung Dis.

[bib-009] Valdés L, San José E, Alvarez D, Sarandeses A, Pose A, Chomón B, Alvarez-Dobaño JM, Salgueiro M, Rodríguez Suárez JR (1993). Diagnosis of tuberculous pleurisy using the biologic parameters adenosine deaminase, lysozyme, and interferon gamma. Chest.

[bib-010] Escudero-Bueno C, Garcia-Clemente M, Cuesta-Castro B (1990). Cytologic and bacteriologic analyzes of fluid and pleural biopsy with Cope’s needle. Arch Intern Med.

